# Use of cleaner-burning biomass stoves and airway macrophage black carbon in Malawian women

**DOI:** 10.1016/j.scitotenv.2018.04.125

**Published:** 2018-09-01

**Authors:** Abigail L. Whitehouse, Lisa Miyashita, Norrice M. Liu, Maia Lesosky, Graham Flitz, Chifundo Ndamala, John R. Balmes, Stephen B. Gordon, Kevin Mortimer, Jonathan Grigg

**Affiliations:** a Centre for Child Health, Blizard Institute, Queen Mary University of London, London, UK; b Division of Epidemiology and Biostatistics, School of Public Health, University of Cape Town, Cape Town, South Africa; c School of Public Health, University of California, Berkeley, Berkeley, USA; d School of Medicine, University of California, San Francisco, San Francisco, USA; e Liverpool School of Tropical Medicine, Liverpool, UK; f Malawi Liverpool Wellcome Trust Programme, Blantyre, Malawi

**Keywords:** Macrophage black carbon, Air pollution, Cookstove, Particulate matter

## Abstract

Exposure to particulate matter (PM) from burning of biomass for cooking is associated with adverse health effects. It is unknown whether or not cleaner burning biomass-fuelled cookstoves reduce the amount of PM inhaled by women compared with traditional open fires.

We sought to assess whether airway macrophage black carbon (AMBC) - a marker of inhaled dose of carbonaceous PM from biomass and fossil fuel combustion - is lower in Malawian women using a cleaner burning biomass-fuelled cookstove compared with those using open fires for cooking. AMBC was assessed in induced sputum samples using image analysis and personal exposure to carbon monoxide (CO) and PM were measured using Aprovecho Indoor Air Pollution meters. A fossil-fuel exposed group of UK women was also studied.

Induced sputum samples were obtained from 57 women from which AMBC was determined in 31. Median AMBC was 6.87 μm^2^ (IQR 4.47–18.5) and 4.37 μm^2^ (IQR 2.57–7.38) in the open fire (*n* = 11) and cleaner burning cookstove groups (*n* = 20), respectively (*p* = 0.028). There was no difference in personal exposure to CO and PM between the two groups. UK women (*n* = 5) had lower AMBC (median 0.89 μm^2^, IQR 0.56–1.13) compared with both Malawi women using traditional cookstoves (*p* < 0.001) and those using cleaner cookstoves (*p* = 0.022).

We conclude that use of a cleaner burning biomass-fuelled cookstove reduces inhaled PM dose in a way that is not necessarily reflected by personal exposure monitoring.

## Highlights

• Sputum induction to assess airway macrophage black carbon is feasible in the field.
• Airway macrophage black carbon represents a way to assess internal dose of particulate matter exposure.
• Cleaner burning biomass-fuelled cookstoves reduce inhaled PM dose.

## Graphical abstract

**Figure:**
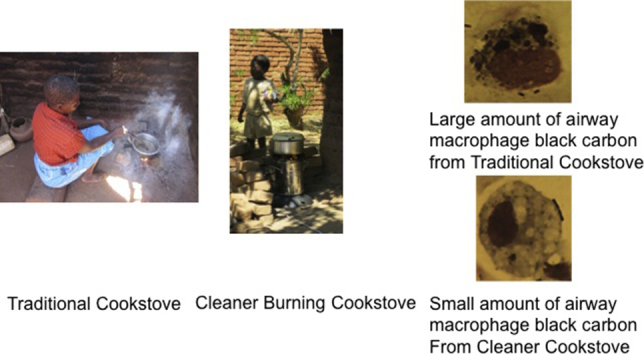

## 1 Introduction

Exposure to carbonaceous particulate matter (PM) from the burning of biomass fuels is associated with a range of adverse health effects, including chronic obstructive pulmonary disease (COPD) in adults (Kelly and Fussell, 2011), and increased risk of pneumonia in infants and young children (Goldizen et al., 2015). Despite robust data from epidemiological studies, interventions aimed at reducing exposure to household air pollution (HAP) have not produced the expected benefits to health. First, in a randomised controlled trial in Guatemala (Randomised Exposure Study of Pollution Indoors and Respiratory Effects, RESPIRE), the provision of a woodstove with a chimney did not reduce physician-diagnosed pneumonia in young children compared with open fire using controls (Smith et al., 2011), albeit severe physician-diagnosed pneumonia was reduced in a secondary analysis. Second, in an recent open cluster randomised study in Malawi (Children and Pneumonia Study, CAPS), we found no difference in rates of pneumonia in young children from households in community clusters assigned to cleaner burning biomass-fuelled cookstoves (Philips HD4012LS; Philips South Africa, Johannesburg, South Africa) compared with continuation of open fire cooking (Mortimer et al., 2017). Possible explanations for this finding include exposure to smoke from other sources including burning of rubbish, tobacco, and income generation activities and exposure from neighbours' cooking fires since cleaner cookstoves were issued only to households that had a resident child younger than 5 years (Mortimer et al., 2017).

An important outstanding question is whether or not use of cleaner burning biomass-fuelled cookstoves reduces inhaled dose of PM in the group most exposed to HAP; i.e. women who do the family cooking. Although measuring long-term personal exposure to PM in adults by portable monitoring is not yet practical, we previously developed a method for assessing inhaled dose of carbonaceous PM by measuring the amount of carbon in airway macrophages (AMBC) obtained using sputum induction. In previous studies, we have found that AMBC is increased in biomass-exposed women in Gondar (Ethiopia) compared to UK women (Kulkarni et al., 2005), and in UK children, found that higher AMBC is associated with impaired lung function (Kulkarni et al., 2006). Although the kinetics of AMBC have not been fully defined, since AM are long-lived cells, AMBC is thought to reflect longer-term exposures (Bai et al., 2014). Since the cookstove used in the CAPS trial reduces PM emissions by about 75% compared to open fires in field tests (Wathore et al., 2017), we hypothesised that AMBC would be reduced in women randomised to the intervention arm of the CAPS trial. We therefore sought to compare AMBC in women using the cleaner cookstove with those using a traditional open fire. We recruited these two groups from women nearing end of the CAPS trial (i.e. after 20–24 months) who were also recruited into the Malawi Adult Lung Health Study (ALHS). In order to give comparison with a non-biomass exposed population a small group of British women were also recruited.

## 2 Methods

This cross-sectional study recruited women from Chikwawa, one of the two sites in rural Malawi used for the CAPS trial. Chikwawa is a district in southern Malawi with a surrounding population of approximately 360,000 people, the majority of whom cook over open fires. We approached women from households included in the CAPS trial who were part of a sub-study called the Adult Lung Health Study (ALHS). ALHS was designed to address the prevalence and determinants of COPD in adults in rural Malawi and the extent to which exposure to HAP explains the rate of decline in lung function (Mortimer, 2017).

Recruitment of women to the study was carried out over 10 days. Before the study, the communication team from the Malawi Liverpool Wellcome Trust's Clinical Research Programme (MLW) visited potential participants to explain sputum induction to identify potential participants at the village level. Twenty villages closest to the Chikhwawa District Hospital that were broadly representative in structure and income of the wider CAPS trial were included. Those that expressed a wish to take part were transported to the Malawi Liverpool Wellcome Research Centre at Chilwawa District Hospital. On arrival, they were provided with group and personal level information, prior to obtaining written consent. Women underwent spirometry (Forced expiratory volume in 1 second, FEV_1_, Forced Vital Capacity, FVC) and sputum induction in accordance with the American Thoracic Society (ATS)/European Respiratory Society (ERS) guidelines (Pizzichini et al., 2002). Women were excluded if they were; i) receiving treatment for active pulmonary tuberculosis, or ii) HIV positive.

The Malawi College of Medicine Research Ethics Committee (Ref P.11/12/1308) and the Liverpool School of Tropical Medicine Research Ethics Committee (Ref (Nwokoro et al., 2012).40) approved the protocol which was peer reviewed and published by The Lancet and is available in open access at www.capstudy.org. Trial registration ISRCTN 59448623 (Mortimer, 2017).

To compare AMBC in Malawian women with women exposed only to fossil fuel PM, we recruited a small group of healthy British women living in London and working at Queen Mary University of London. They were approached by the research team with written information and completed sputum inductions after written consent was obtained. The same team who did the sampling in Malawi carried out the sputum induction and processing in the UK. Ethical approval for UK controls was granted by HRA NRES Centre Manchester REC committee 13/LO/0440.

Sputum induction was done using a standardised technique using nebulised hypertonic saline (3.5% for a maximum of 20 min) (Pizzichini et al., 2002). Induced sputum samples were placed on ice, and transported to the University of Malawi, College of Medicine, Blantyre, for processing within 4 hours. In the UK sputum induction was done onsite at the Royal London Hospital and samples were placed on ice and processed within 4 hours. Specimens from Malawi and the UK were processed identically. Briefly, mucolysis was first carried out by vortexing in the presence of 0.1% Dithiothreitol, then cells are cytospun as previously described (Kulkarni et al., 2006). Slides were imaged by light microscopy at ×100 magnification in oil (Moticam1000 camera, Motic Europe or Mazurek Optical Services microscope and camera), digital images transferred to ImageJ software, and analysed for AMBC as previously described. Briefly, digital images of 50 randomly selected AM were analysed for AMBC and data expressed as mean area per AMBC per subject (μm^2^) (Kulkarni et al., 2006; Brugha et al., 2015; Nwokoro et al., 2012).

Personal exposures of Malawian women to CO in mean ppm and fine particulate matter (PM_2.5_) in μg/m^3^ were measured over a 48-h period as part of the ALHS study using Aprovecho Indoor Air Pollution meters (Aprovecho Research Centre, OR, USA). Monitoring of CO and PM_2.5_ was done once the intervention cookstoves were in place and at least one year before assessment of AMBC and are indicative of average exposures over the study time-period.

## 3 Study power and statistical analysis

From our previous AMBC data (Kulkarni et al., 2005), recruitment of 18 subjects in the traditional cookstove and 18 intervention cookstove groups had a power to detect a 50% difference in mean AMBC at 5% significance and 80% power. Data are summarised as median (IQR) and compared by Mann Whitney U test. Age and lung function is summarised by mean (SD) and compared by *t*-test. Statistical analysis was carried out using GraphPad Prism version 6 (GraphPad Software, La Jolla, USA).

## 4 Results

**Fig. 1:**
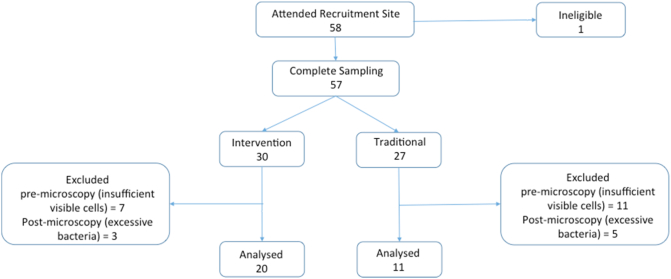
Flow chart of recruitment to study and samples analysed.

We recruited 58 (30 intervention with cleaner burning cookstoves and 28 control with traditional cookstoves) women (range 16 to 45 y) (Fig. 1), Table 1). One potential participant was excluded as she had failed to disclose her HIV status prior to the sampling. Five healthy non-smoking women were recruited in the UK (Caucasian *n* = 3, Asian *n* = 2), all lived and worked in central London, cooked using gas, and commuted to work by public transport (train/tube/bus) or by cycle.

**Table 1:** Baseline demographic data. Groups are compared by *t*-test and reported as Mean (SD), apart from measured exposures which are compared by Mann Whitney and expressed as Median (IQR).

n	Traditional	Intervention	*p*
	28	30	
Age (y)	30.4 (8.3)	30.5 (6.7)	0.98
FEV_1_% predicted^	85.88 (10.39)	88.40 (10.28)	0.43
FVC % predicted^	87.62 (11.88)	89.23 (9.6)	0.63
FEV_1_:FVC ratio^	0.86 (0.06)	0.86 (0.05)	0.88
CO (ppm)+	1.35 (0.6–1.68)	1.25 (0.8–1.9)	0.81
PM_2.5_ (μg/m^3^)+	63.15 (33.83–86.28)	80.90 (49.94–124.9)	0.16

AMBC, airway macrophage black carbon. CO, carbon monoxide. PM_2.5_, particulate matter <2.5 μm. FEV_1_, forced expiratory volume in 1 s. FVC, forced vital capacity.

+ Variables compared using Mann Whitney.

^ Variables compared using students t-test.

Age, FEV_1_, FVC predicted and FEV_1_/FVC were similar between the two Malawian groups (Table 1).

**Fig. 2:**
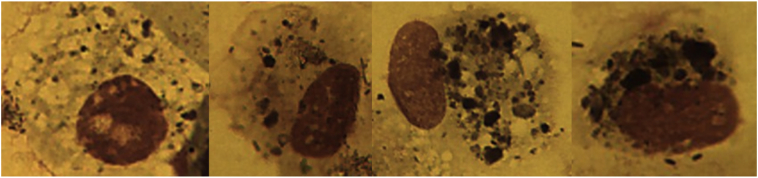
Airway macrophages from the samples obtained in Malawi demonstrating large amounts of visible black carbon in clumps (airway macrophage black carbon, AMBC) (x100 under oil immersion).

**Fig. 3:**
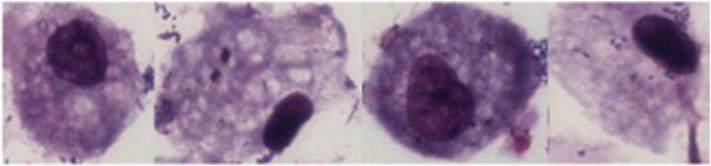
Airway macrophages from the UK samples showing some large clumps of black carbon along with smaller scattered visible particles (airway macrophage black carbon, AMBC) (x100 under oil immersion).

Sputum induction was done in 58 Malawian women, and 5 British women. Aggregates of carbonaceous PM were visible in AMs from all Malawian women, with some cells exhibiting particularly high levels of carbon loading (Fig. 2). In contrast, high carbon loading was not seen in AM from any of the British women (Fig. 3).

**Fig. 4:**
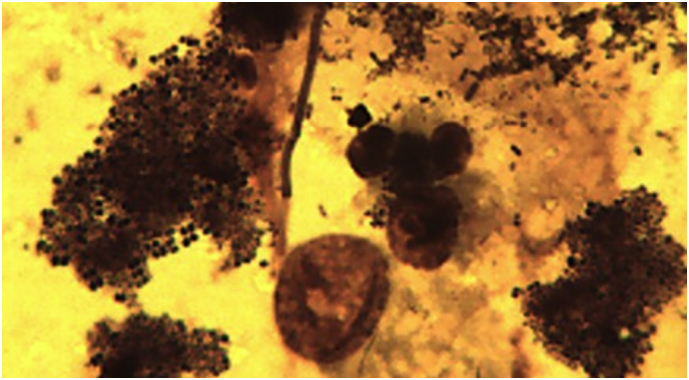
Microscope image demonstrating large sheets of bacteria obscuring the macrophages (x100 under oil immersion).

**Fig. 5:**
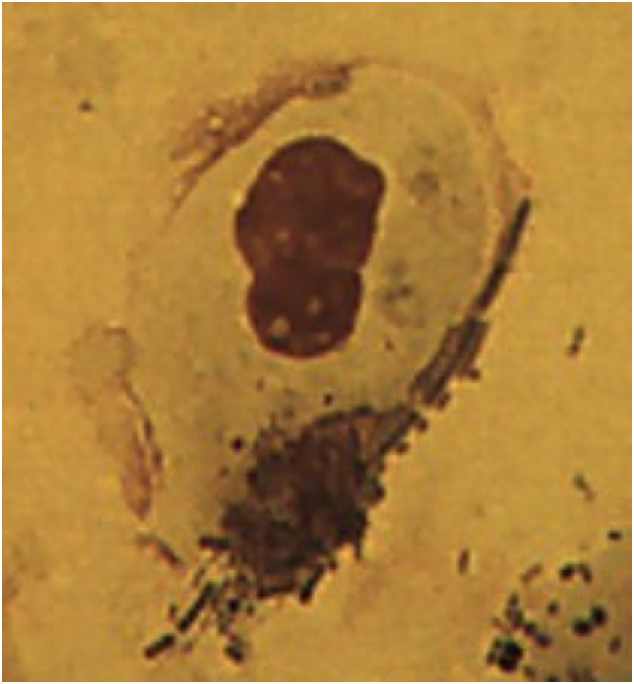
Single macrophage with phagocytosed bacteria (x100 under oil immersion).

Induced sputum samples from 26 (10 intervention; 16 control) Malawian women had either too few AM to calculate AMBC (*n* = 18) or contained large sheets of bacteria that obscured induced AMs (*n* = 8, Fig. 4). The few AM that could be visualised under light microscopy in women with bacterial sheets contained phagocytosed bacteria (Fig. 5). Since the upper airway does not contain AM, this observation suggests that the bacteria seen originated from the lower airway. There was no significant difference in the baseline characteristics of the participants that had samples suitable for AMBC analysis compared to those that were not (Table 2). None of the samples from British women had bacterial sheets.

**Table 2:** Comparison of Malawi women in whom AMBC either was or was not determined Groups are compared by *t*-test and reported as Mean (SD).

n	AMBC determined	AMBC not determined	*p*
	31	27	
Age (y)	30.2 (7.87)	30.7 (7.11)	0.83
FEV_1_% predicted	85.01 (10.9)	88.99 (9.6)	0.21
FVC % predicted	90.37 (10.36)	86.09 (10.74)	0.19
FEV_1_:FVC ratio	0.85 (0.06)	0.85 (0.05)	0.94

SD, standard deviation. FEV_1_, forced expiratory volume in 1 s. FVC, forced vital capacity.

**Fig. 6:**
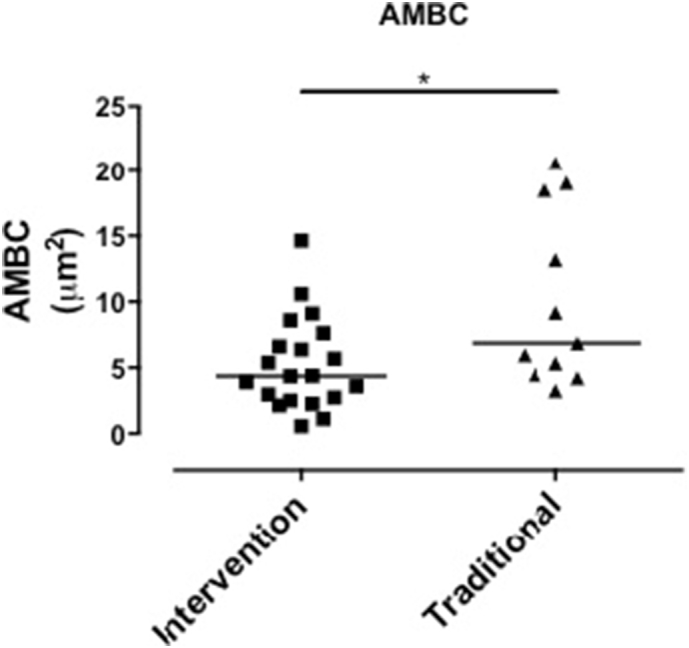
Comparison of Airway macrophage black carbon results between the traditional cookstove group and the intervention cleaner cookstove group, each dot represents a separate individuals mean AMBC (per 50 macrophages), *p* < 0.05 by Mann Whitney.

**Fig. 7:**
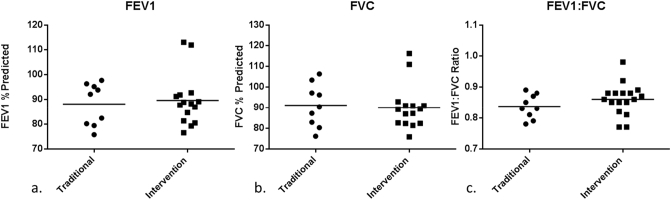
Dotplots comparing AMBC subgroup lung function data, each dot represents a single participants result.. a. shows % predicted FEV_1_. b. shows % predicted FVC. c. shows FEV_1_:FVC ratio. FEV_1_, forced expiratory volume in 1 s. FVC, forced vital capacity.

Malawian women in the intervention group (*n* = 20) had lower AMBC compared to those in the control group (*n* = 11); median 4.37 μm^2^ (IQR; 2.57 to 7.38) vs. 6.87 μm^2^ (4.47 to 18.5), *p* = 0.028 (Table 3, Fig. 6). There were no differences between intervention and controls groups in lung function, personal 48-h CO or PM_2.5_ exposure (Table 3, Fig. 7). Furthermore there were no differences in lung function, personal 48-h CO or PM_2.5_ exposure between women in the intervention and traditional groups when analyses included all women who took part in the study, i.e. not only those in whom AM carbon was determined (Table 1).

**Table 3:** AMBC, monitored exposure, and lung function on subgroup of Malawian women with AMBC determined.

n	Traditional	Intervention	*p*
	11	20	
AMBC (μm^2^)+	6.88 (4.48–18.5)	4.37 (2.57–7.37)	0.028^⁎^
CO (ppm)+	1.6 (1.45–2.85)	1.5 (0.9–2.1)	0.45
PM_2.5_ (μg/m^3^)+	46 (32.5–103)	89 (53–128)	0.16
FEV_1_% predicted^	88.05 (8.5)	89.54 (10.4)	0.72
FVC % predicted^	91.07 (10.4)	89.95 (10.68)	0.80
FEV_1_:FVC ratio^	0.83 (0.04)	0.86 (0.05)	0.24

AMBC, airway macrophage black carbon. CO, carbon monoxide. PM_2.5_, particulate matter <2.5 μm. FEV_1_, forced expiratory volume in 1 s. FVC, forced vital capacity.

+ Variables compared by Mann Whitney test.

^ Variables compared by students t-test.

**Fig. 8:**
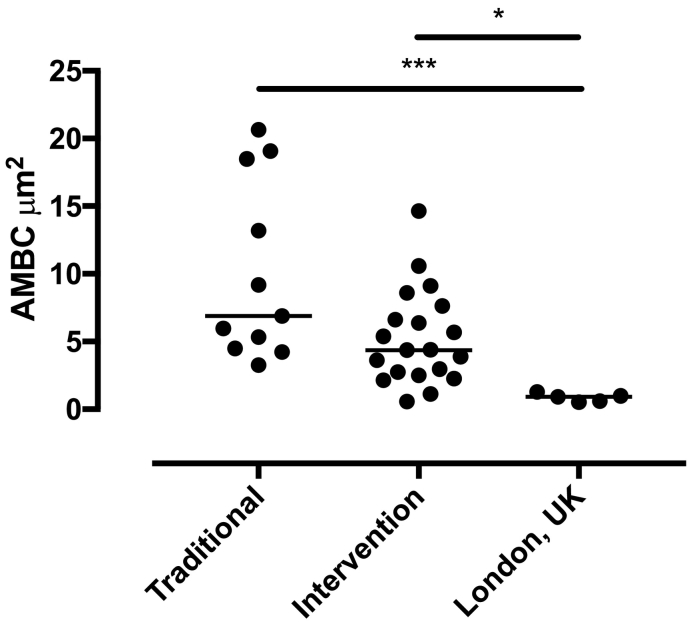
Comparison of Airway macrophage black carbon results between the traditional cookstove group and the intervention cleaner cookstove group and the healthy UK controls, each dot represents a separate individuals mean AMBC (per 50 macrophages), *p* < 0.05 by Kruskall Wallis and post hoc testing.

Malawian women (combined) had significantly higher AMBC compared with British women; 5.38 μm^2^ (IQR 3.3 to 9.1) vs. 0.89 μm^2^ (IQR 0.56–1.12), *p* = 0.0006 (Table 4, Fig. 8).

**Table 4:** AMBC in samples from Malawi compared to UK. Compared by Kruskall Wallis and post-hoc to compare the Malawian groups and British controls.

n	Malawian traditional	Malawian intervention	British controls
	11	20	5
AMBC (μm^2^)	6.88 (4.48–18.5)+	4.37 (2.57–7.37)^	0.89 (0.56–1.12)

+ Traditional vs UK p = 0.0004**.

^ Intervention vs UK p = 0.022*.

**Fig. 9:**
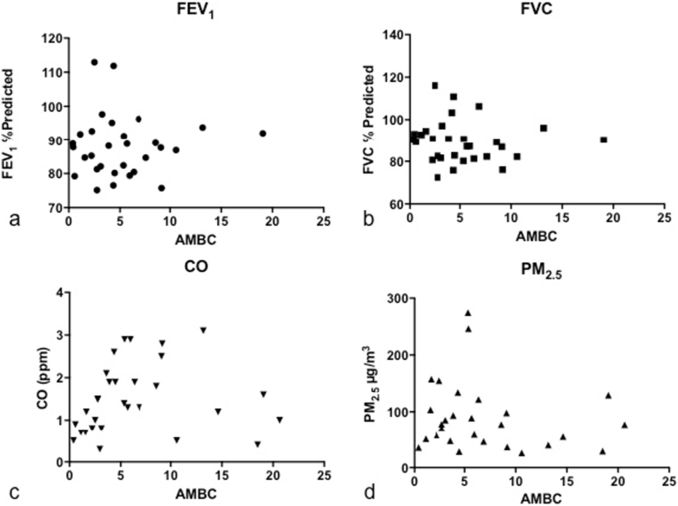
Individual scatter plots of mean AMBC against; a. FEV_1_% Predicted, b. FVC% Predicted, c. PM_2.5_, d. CO. All correlations are non-significant (see Table 5). FEV_1_, forced expiratory volume in 1 s. FVC, forced vital capacity. AMBC, airway macrophage black carbon (mean per 50 macrophages per subject). CO, carbon monoxide. PM_2.5_, particulate matter <2.5 μm.

There was no significant correlation between AMBC and either the lung function variables or the exposure variables (Table 5, Fig. 9).

**Table 5:** Correlation between AMBC and FEV_1_, FVC, CO and PM_2.5_.

	FEV_1_% predicted	FVC % predicted	CO	PM_2.5_
r_s_	0.016	−0.20	0.34	−0.198
p	0.93	0.28	0.06	0.31

Analysed by Spearman's correlation.

## 5 Discussion

This is the first study of the effect of a cleaner burning biomass-fuelled cookstove on inhaled dose of carbonaceous PM. We found that Malawian women using the cleaner cookstove had 36% lower AMBC compared with those who used an open fire for cooking. This is consistent with our previous bronchoalveolar lavage study in Malawi, which found that AMBC reflects the fuel used for heating and lighting in the home (Fullerton et al., 2009). For example, we found highest AMBC in subjects using tin lamps for lighting, and the lowest AMBC in those who used electric lighting. The present study suggests that, in individuals who are regularly exposed to high levels of PM emitted from the burning of biomass, use of a cleaner burning cookstove can reduce exposures which may in turn result in health benefits. Although the nature and extent of these benefits remain unclear, we previously reported that in vivo AM carbon particulate loading is inversely related to capacity to produce an effective antibacterial response (Rylance et al., 2015) and thus speculate that reduced AMBC loading from use of a cleaner cookstove reduces the risk of lower respiratory tract infection. In addition, since chronic exposure to biomass smoke is associated with lung function changes that are compatible with chronic obstructive airways disease, significant reductions in inhaled PM dose from cleaner cookstoves may attenuate the accelerated lung function decline thought to occur in this population of women (Montes de Oca et al., 2017).

The observation of sheets of free bacteria in the induced sputum of a subset of Malawian women was an unexpected finding, since these women were free of respiratory symptoms. Indeed, we have never observed this phenomenon in our extensive experience analysing induced sputum samples from UK subjects. Although an upper airway origin for these bacteria cannot be excluded, a lower airway origin is most likely since; i) all women rinsed their mouth and blew their nose prior to induction, and ii) we observed AM phagocytosis of bacteria in subjects with bacterial sheets. Whether excessive free bacteria reflects, as previously discussed, PM-induced impairment of host immune defence is unclear, but this observation is consistent with the recent study by Rylance et al. (2016) who found increased abundance of *Neisseria* and *Streptococcus* in bronchoalveolar lavage samples from apparently healthy Malawian adults who were exposed to high concentrations of PM. The prevalence and mechanisms for persistent bacterial lower airway colonisation, and alterations of the airway microbiome in biomass-exposed population therefore merits further study.

The marked overlap in AMBC between the two Malawian groups, is compatible with our observation reported in the CAPS trial paper (Mortimer et al., 2017) that there are other major sources of exposure to carbonaceous PM in this population. For example, women regularly visit other homes, there is frequent open burning of rubbish in villages, and women walk alongside roads where traffic, albeit light, is dominated by diesel cars and trucks with considerable exhaust emissions. Exposure to these other sources may explain why no difference in 48-h personal CO/PM_2.5_ exposure was found. But the reason for the discrepancy between AMBC and short-term monitored exposure is unclear. We speculate that one explanation is that high peak exposures to biomass PM have a disproportionate effect on inhaled dose, and thus AMBC. Indeed, we previously observed a disproportionate effect of PM peaks on AMBC in a study of two groups of London commuters who were either cycling or walking to work. In this previous study, we found that, although overall 24-h monitored black carbon was not significantly different between the two groups, monitored black carbon during the commute was higher in cyclists, AMBC was higher in cyclists, and there was an association between monitored black carbon peaks and AMBC (Nwokoro et al., 2012; Nwokoro et al., 2013).

A limitation of this study is that, due to the short time period available for sampling by the UK research team, the study is underpowered for other secondary outcome of potential interest. For example, in a group of 65 healthy young people living in Leicester (UK) exposed to fossil-fuel emission, we previously found a significant inverse correlation between FEV_1_ and AMBC (Kulkarni et al., 2006). It would therefore be of interest to assess this inverse association in a larger, and adequately powered, study of young people whose AMBC is predominately from exposure to biomass smoke.

In summary, we found direct evidence that use of cleaner burning biomass-fuelled cookstoves by women reduced the inhaled dose of carbonaceous PM. We also demonstrated new insights into the possibility of higher bacterial load in lower airway samples than previously thought, this is important when considering the implications for higher pneumonia incidence. We found that it is feasible to induce sputum samples in the field, and subsequently transport and process samples for advanced mechanistic studies. We therefore conclude that use of sputum induction in future studies will provide important insights into the development of respiratory disease in rural populations in low-income countries.

## Funding

The ALHS within which this work was nested was funded by:

New Investigator Research Grant (Mortimer) from the Medical Research Council (Ref: MR/L002515/1).

National Institute for Environmental Health Sciences (US) grant (Balmes and Mortimer) (R56 ES023566)

The ALHS was one of the research themes of CAPS funded by:

Joint Global Health Trials Grant from the Medical Research Council (Ref: MR/K006533/1), UK Department for International Development and Wellcome Trust (Ref: 085790/Z/08/Z).

Additional support was provided by a MRC Partnership Grant “BREATHE-AFRCA” (Ref: MR/L009242/1) and the Malawi Liverpool Wellcome Trust Programme of Clinical Research (Ref: Wellcome Trust 206545).

## References

[bb0005] Bai Y. (2014). Carbon loading in airway macrophages as a biomarker for individual exposure to particulate matter air pollution - a critical review. Environ. Int..

[bb0010] Brugha R. (2015). Respiratory tract dendritic cells in paediatric asthma. Clin. Exp. Allergy.

[bb0015] Fullerton D.G. (2009). Domestic smoke exposure is associated with alveolar macrophage particulate load. Tropical Med. Int. Health.

[bb0020] Goldizen F.C., Sly P.D., Knibbs L.D. (2015). Respiratory effects of air pollution on children. Pediatr. Pulmonol..

[bb0025] Kelly F.J., Fussell J.C. (2011). Air pollution and airway disease. Clin. Exp. Allergy.

[bb0030] Kulkarni N.S. (2005). Carbon loading of alveolar macrophages in adults and children exposed to biomass smoke particles. Sci. Total Environ..

[bb0035] Kulkarni N. (2006). Carbon in airway macrophages and lung function in children. N. Engl. J. Med..

[bb0040] Montes de Oca M. (2017). Smoke, biomass exposure, and COPD risk in the primary care setting: the PUMA study. Respir. Care.

[bb0045] Mortimer, K. Protocol 13PRT/4689: an Advanced Cookstove Intervention to Prevent Pneumonia in Children Younger than 5 years in Malawi: a Cluster Randomised Controlled Trial (ISRCTN59448623). 2017 (31/01/2017); (Available from): http://www.thelancet.com/protocol-reviews/13PRT-4689.

[bb0050] Mortimer K. (2017). A cleaner burning biomass-fuelled cookstove intervention to prevent pneumonia in children under 5 years old in rural Malawi (the cooking and pneumonia study): a cluster randomised controlled trial. Lancet.

[bb0060] Nwokoro C. (2012). Cycling to work in London and inhaled dose of black carbon. Eur. Respir. J..

[bb0065] Nwokoro C., Brugha R., Grigg J. (2013). Alveolar macrophages carbon load: a marker of exposure?. Eur. Respir. J..

[bb0070] Pizzichini E. (2002). Safety of sputum induction. Eur. Respir. J..

[bb0090] Rylance J. (2015). Household air pollution causes dose-dependent inflammation and altered phagocytosis in human macrophages. Am. J. Respir. Cell Mol. Biol.

[bb0075] Rylance J. (2016). Household air pollution and the lung microbiome of healthy adults in Malawi: a cross-sectional study. BMC Microbiol..

[bb0080] Smith K.R. (2011). Effect of reduction in household air pollution on childhood pneumonia in Guatemala (RESPIRE): a randomised controlled trial. Lancet.

[bb0085] Wathore R., Mortimer K., Grieshop A.P. (2017). In-use emissions and estimated impacts of traditional, natural- and forced-draft Cookstoves in rural Malawi. Environ. Sci. Technol..

